# Asymptomatic Spontaneous Migration of the Tip of Port-A-Cath System Into the Right Internal Jugular Vein: A Case Report of an Uncommon Complication

**DOI:** 10.7759/cureus.26937

**Published:** 2022-07-17

**Authors:** Dimitrios Diamantidis, Nikolaos Papatheodorou, Sempachedin Perente, Sotirios Botaitis

**Affiliations:** 1 1st General Surgery Department, University Hospital of Alexandroupolis, Alexandroupolis, GRC

**Keywords:** catheter migration, internal jugular vein, complication, spontaneous migration, venous port catheter

## Abstract

Venous port catheters are devices that allow access to the central venous system and, in clinical practice, are used for patients who require long-term intravenous therapy. The ideal position of the catheter tip is the distal superior vena cava and can be confirmed by a postoperative chest X-ray. Complications during and after the implantation are not rare, but spontaneous migration of the catheter tip into the internal jugular vein is an uncommon complication. Catheter migration may be accompanied by neck, shoulder, and ear pain. Venous phlebitis and thrombosis, and neurological complications, can become potentially life-threatening. We report a case of a spontaneous catheter tip migration into the right internal jugular vein that was diagnosed in a random chest roentgenography. The patient was taken to the operative room, and the catheter was successfully removed.

## Introduction

Port-A-Cath systems have been extensively used since 1982 when Niederhuber [1] proposed the implantation of venous port catheters for patients with cancer. Oncologic patients require long-term intravenous therapy, and central venous port catheters provide a reliable access for chemotherapy, parenteral nutrition, infusions, or blood transfusions [2,3]. The overall complication rate of venous port systems is estimated to be 7.2%-12.5%, with spontaneous migration being an uncommon delayed complication, with an incidence of about 0.1%-1.8% [2,4]. We present a case of an asymptomatic, spontaneous migration of a port catheter into the right jugular vein.

## Case presentation

**Figure 1:**
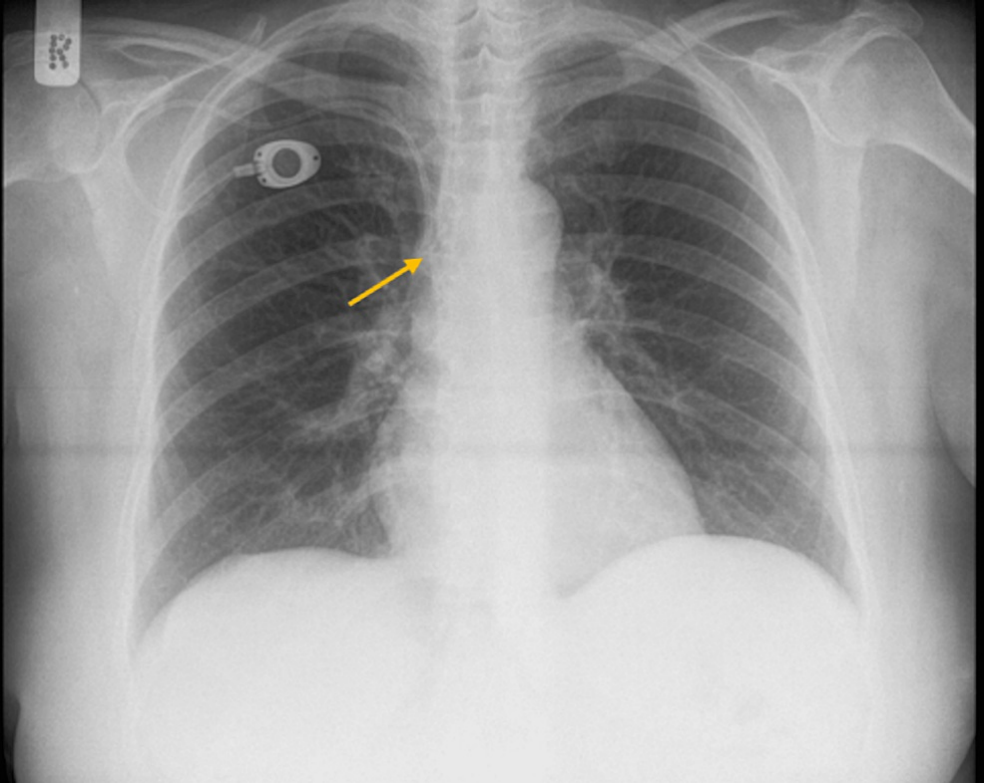
Chest X-ray after the implantation showing the position of the catheter tip in the upper portion of the superior vena cava

**Figure 2:**
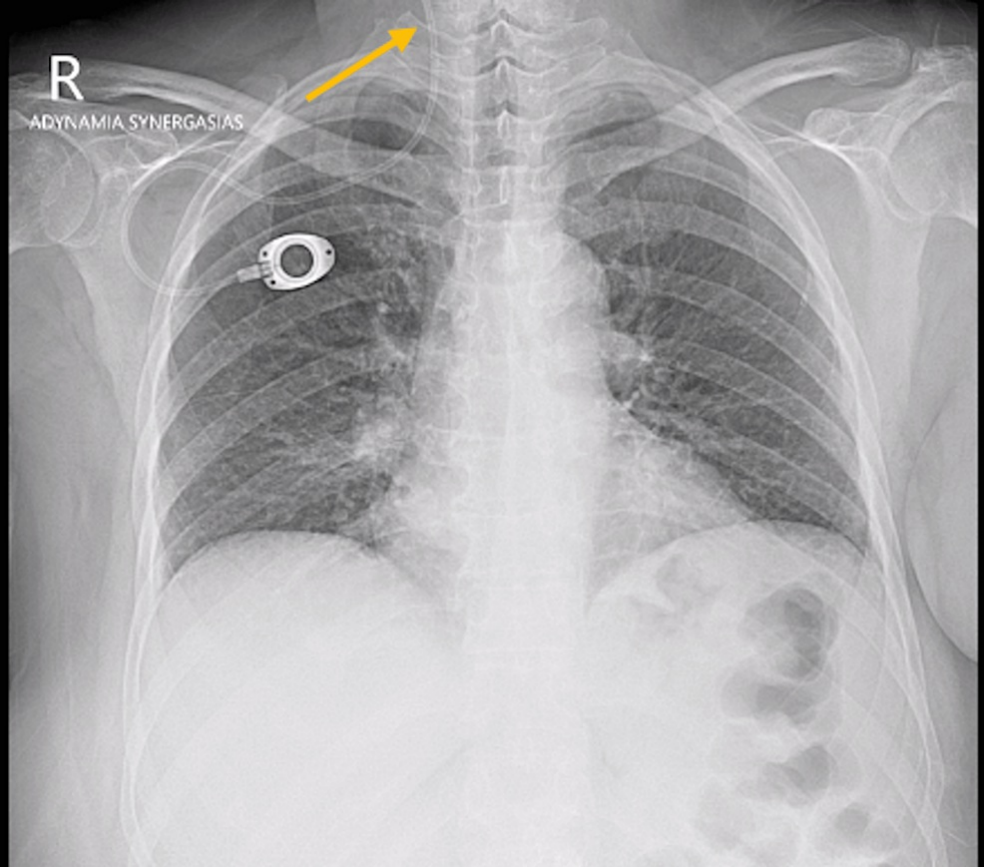
Chest X-ray showing the distal tip of the catheter into the right internal jugular vein

A 57-year-old woman with a body mass index (BMI) of 35.3 who was diagnosed with advanced breast cancer received implantation of a Port-A-Cath for the courses of chemotherapy after she underwent a modified radical mastectomy. The implantation of the catheter was done under local anesthesia via the right subclavian vein, using Seldinger’s technique. After the implantation, the position of the catheter tip in the upper portion of the superior vena cava (SVC) was confirmed by postoperative chest roentgenography (Figure 1). Afterward, she received the cycles of chemotherapy without any complications, but then the patient was lost to follow-up. Three years after the implantation, she had to be hospitalized and operated on for a non-reducible abdominal hernia. As part of the preoperative examination, on the chest radiograph, migration of the catheter tip to the internal jugular vein was observed (Figure 2). According to her medical history, the patient did not experience any symptoms during this period. The patient was taken to the operative room, and before the surgical repair of the abdominal hernia the catheter was removed. The catheter was not replaced, as the course of chemotherapy for the initial diagnosis of breast cancer had been deemed successful, and no further long-term intravenous therapy was required.

## Discussion

Venous port catheters are devices that allow access to the central venous system, and since 1982, when Niederhuber [1] proposed the implantation of port catheters for patients with cancer, they are widely used in clinical practice. These devices are suitable for patients who require long-term intravenous therapy, and they provide reliable access for the delivery of chemotherapeutic-cytotoxic drugs, parenteral nutrition, infusions, or blood transfusions [3,5]. The overall complication rate of venous port systems is estimated to be 7.2%-12.5% and complications could be divided into early (appear in less than 30 days after implantation) and delayed (appear more than 30 days after implantation) [2].

Pneumothorax, hemothorax, thoracic duct injury, air embolism, and catheter malposition are early complications, and based on severity, pneumothorax and hemothorax are the most likely major complications. Delayed involves venous thrombosis, infection, pulmonary embolism, catheter fracture, and migration. Spontaneous migration of the port catheter is an uncommon complication following long-term access to the central venous system, with an incidence of about 0.1%-1.8% [2,4].

In the literature, 11 patients were reported with spontaneous migration of a venous port catheter. The indication for implantation of a Port-A-Cath was breast cancer in three patients, lung cancer in three patients, cystic fibrosis in two patients, prostate cancer in one patient, acquired immunodeficiency syndrome in one patient, and transfusion in one patient [6-11]. Our patient was diagnosed with advanced breast cancer and required the implantation of a venous port catheter for the cycles of chemotherapy after she underwent a modified radical mastectomy.

Catheter tip migration may be accompanied by various symptoms, such as the neck, shoulder, and ear pain. Additionally, neurological complications, venous phlebitis, and thrombosis could be potentially life-threatening, when the catheter is used to infuse drugs [6]. Among the patients from the previous studies, three proceeded to the hospital with right neck pain, three with thrombophlebitis, and five were asymptomatic [6-11]. In our case, the migration of the catheter was diagnosed on random imaging, and the patient did not experience any symptoms after implantation, or during the maintenance period. 

The mechanism for spontaneous migration is yet not clear, and several factors may contribute. Changes in the pressure within the thoracic cavity due to weight lifting, coughing, sneezing, and straining are proposed to induce tip migration. Additionally, physical movement, changes in the body position, neck flexion, congestive heart failure, and high-flow infusion rate may cause such migration [6,12,13]. Risk factors such as obesity and female gender did not appear to be associated [14].

To prevent further complications, potentially life-threatening, the management of such complications is either to remove or to replace the migrated catheter. The removal can be proposed if the patient does not require long-term intravenous therapy. Otherwise, replacement is recommended [3,11]. Catheter repositioning into the SVC via a transfemoral approach can be attempted if the initial position of the tip was ideal [6]. In this case, the patient did no longer require long-term intravenous therapy, as the course of chemotherapy for the initial diagnosis of breast cancer had been deemed successful. Therefore, the removal of the venous port catheter was decided.

## Conclusions

In conclusion, spontaneous migration of a Port-A-Cath system is an uncommon complication. Several risk factors are proposed to induce the migration of the tip, and the patient may suffer from various symptoms, some of them potentially life-threatening. Therefore, awareness of this possibility is important, and a regular review of the catheter tip on chest roentgenography images is suggested. As there are no distinct guidelines in the literature regarding the proper diagnosis of port catheter migration, we recommend that the position of the catheter should always be determined before the infusion, by a chest X-ray. Additionally, when the venous port system is not in use, the patient should monitor the position every six months, otherwise, the removal is suggested. In the case of a migrated catheter, the clinicians should decide either to remove or replace the venous port system.
